# Instrumented Indentation of Super-Insulating Silica Compacts

**DOI:** 10.3390/ma12050830

**Published:** 2019-03-12

**Authors:** Belynda Benane, Sylvain Meille, Geneviève Foray, Bernard Yrieix, Christian Olagnon

**Affiliations:** 1 Univ Lyon, INSA-Lyon, MATEIS, UMR CNRS 5510—7 avenue Jean Capelle, F-69621 Villeurbanne, France; belynda-b.benane@edf.fr (B.B.); genevieve.foray@insa-lyon.fr (G.F.); christian.olagnon@insa-lyon.fr (C.O.); 2 Univ Lyon, INSA-Lyon, CNRS, UCBL, MATEB, 7 avenue Jean Capelle, F-69621 Villeurbanne, France; bernard.yrieix@edf.fr; 3 EDF R&D, Les Renardières, F-77250 Moret sur Loing, France

**Keywords:** silica, super-insulating materials, instrumented indentation, porosity

## Abstract

Highly porous silica compacts for superinsulation were characterized by instrumented indentation. Samples showed a multi-scale stacking of silica particles with a total porous fraction of 90 vol %. The two main sources of silica available for the superinsulation market were considered: fumed silica and precipitated silica. The compacts processed with these two silica displayed different mechanical properties at a similar porosity fraction, thus leading to different usage properties, as the superinsulation market requires sufficient mechanical properties at the lowest density. The measurement of Young’s modulus and hardness was possible with spherical indentation, which is an efficient method for characterizing highly porous structures. Comparison of the mechanical parameters measured on silica compacts and silica aerogels available from the literature was made. Differences in mechanical properties between fumed and precipitated compacts were explained by structural organization.

## 1. Introduction

Reducing energy consumption in buildings is a critical issue, as it represents more than 40% of the total energy consumption in the so-called developed countries [1,2]. To drastically reduce the energy needed for heating and cooling purposes in buildings, new classes of super-insulating materials are needed, allowing either to ensure a higher insulation capacity as compared to standard insulation materials or reduce the current thicknesses of insulating materials at a given insulation capacity. The most promising solution is currently vacuum insulation panels (VIP), which are made of a core of slightly compacted silica nanopowders (with typically 90% of porosity) wrapped into a sealed membrane under vacuum. Thermal conductivities as low as 2 to 5 mW/(m·K) can be achieved for these structures, which is eight times lower than conventional insulation materials such as expanded polystyrene or mineral wool [3]. However, a major limitation to the commercial development of VIP is their high price. To reduce it, the preferential solution would be to replace the fumed silica (FS) that is currently used as the core material with precipitated silica (PS), which is largely used in many industrial applications (tires, pharmaceutics, etc.). The former are synthesized at high temperature, the latter in aqueous solution at a lower price and in larger quantities. The choice of the nature of the silica nanopowder has a major influence on the mechanical characteristics and on the thermal conductivity of VIP [4,5,6]. At a given compaction pressure, PS compacts are denser and therefore have a higher thermal conductivity as compared with FS compacts [7], thus limiting the development of VIP with a core made with PS. Therefore, it is of critical importance to understand the origins of such differences in the mechanical behavior between compacts of PS and FS powders. This could open new avenues to either develop more competitive solutions in superinsulation or improve existing solutions.

The structural characteristics of PS silica nanopowders have been largely studied using small angles X-ray scattering (SAXS) [8,9] and transmission electronic microscopy (TEM) [10]. It was shown that these powders have a pronounced multi-scale structure that is made of elementary silica nanoparticles (5 to 20 nm in diameter) organized at larger scales in aggregates (in the 100 to 200-nm size range) and agglomerates (400 to 500-nm size range). The nanometer pore size combined with the very open structure of the silica and the presence of free dangling arms limit the thermal conduction through the gas and the solid phases. FS powder and compacts were recently studied using SAXS, TEM, and mercury instruction porosimetry [11]. The multi-scale organization already noted for PS was confirmed. Moreover, the non-spherical shape of aggregates of FS particles (necklace-shaped and disk-shaped) at the submicron-length scale was highlighted.

Whereas numerous publications deal with the mechanical properties of highly porous ceramics and glasses in the range of 50% to 75% of porosity [12,13,14,15,16,17,18,19], the literature offers a limited number of papers dealing with the mechanical properties of highly porous mineral materials. Most studies deal with the mechanical properties of highly porous silica in the form of aerogels that are used as super-insulating material at atmospheric pressure, with a range of density from 0.08 to 0.35 g/cm^3^, i.e., 4% to 15% of solid fraction, which is similar to or lower than that for silica compacts (typically 10%) [20,21]. Silica aerogels have been characterized in terms of their elastic modulus, compression strength, and fracture toughness for different apparent densities [22,23]. The preparation of samples for standard mechanical testing was noted as delicate in such materials with very low mechanical properties [21,23]. Instrumented indentation has also been used to characterize the properties of silica aerogels, namely elastic modulus and hardness. Typical values of elastic modulus and hardness depend on the solid content, *E,* varying from 1 to 100 MPa, and *H* varying from 0.1 to 10 MPa for solid fractions ranging from 5 to 20 vol % [21,23]. Ultra-low density aerogels (apparent density below 0.04 g/cm^3^, i.e., 2% solid fraction) have also been characterized [21,24]. Their mechanical behavior in spherical indentation tends to be mainly elastic with a large deformation capacity [25]. In addition to the influence of the structure on properties, an influence of the relative humidity (RH) on the mechanical behavior of highly porous silica aerogels has also been noted, with an increase of the time-dependent contribution at high RH [26]. Silica powders in the form of dense compacts for pharmaceuticals have been studied, but at relative densities above 30%, which is much larger than the density that is needed for the superinsulation market [27]. To our knowledge, no work has been published on the mechanical properties of silica compacts with densities in the range of superinsulation materials.

This work focuses on the characterization of the mechanical properties of compacts of nanostructured silica powders to be used as the core material of VIP. Two nature of silica were considered: fumed silica (FS) and precipitated silica (PS). Due to the high volume fraction of the porosity in such structures, their mechanical properties are very low, and the preparation of samples with well-defined geometries for standard tests to characterize mechanical properties (flexion, compression) is delicate. Uniaxial compression strength is also dependent on the aspect ratio of samples, as the friction between platens and sample surface can influence the measurement of strength. Instrumented indentation offers a credible alternative due to its relative easy setup, quasi-nondestructive character, and the ability to run several tests on a single sample.

Indentation tests were carried out with sharp and spherical tips at a large penetration depth to test a large volume of material, allowing averaging the contributions of both solid and porous phases in the material [28,29,30,31,32]. Test were carried out on both FS and PS silica compacts at the same total porosity fraction to help understand the differences in the properties noted in VIP formulations between the two sources of silica. All of the tests were performed at ambient conditions to limit the influence of water absorption on the properties of materials.

## 2. Materials and Methods 

Two commercial nanostructured silica powders were characterized—a fumed silica (Konasil 200, OCI, Seoul, Korea) and a precipitated silica (T43, Solvay, Collonges-au-Mont-d’Or, France)—both with purity above 99.5%. Cylindrical pellets of FS and of PS powders were fabricated by oedometric compression on a Zwick testing machine (BZ1-MM1195, Zwick-Roell, Ulm, Germany) equipped with a steel die (inner diameter of 20 mm) and a brass piston. The same weight of FS or PS powder (200 mg) was introduced in the die before compaction. The surface roughness of the sample was controlled by a disk of mirror-polished sapphire (20-mm diameter and 2-mm thick) placed inside the die below the silica compact. The crosshead speed during loading and unloading were 10 mm·mn^−1^ and 5 mm·mn^−1^ respectively; a one-hour stress relaxation stage was made after the loading phase, as it promotes the flattening of the sample’s surface. A typical roughness *Ra* of 1.5 µm was measured on the tested sample’s surface using a Hirox KH-7700 digital microscope (Hirox-Europe, Limonest, France). A similar volume fraction of pores was targeted for both FS and PS silica samples for the sake of comparison in mechanical properties (10% of solid volume fraction targeted, i.e., an apparent density of 210–220 g/m^3^). To reach the targeted apparent densities after compaction, maximum applied pressures were 0.6 MPa and 0.15 MPa for FS and PS respectively, illustrating the easier densification of PS powder as compared with FS powders. The final sample diameter and height were 20 mm and 3 mm, respectively. All of the compaction tests were performed at 20 °C and between 30–45% RH (measured by a thermohygrometer, Testo 625, Testo, Lenzkirch, Germany) to limit the influence of environmental conditions. Table 1 gathers the main physical parameters measured on FS and PS silica powders and on FS and PS pellets.

Instrumented indentation tests were performed on a nanoindenter G200 (Agilient, Santa Clara, CA, USA), equipped with a continuous stiffness measurement (CSM) module. Synthetic sapphire spherical indenter tips were used with diameters of 600 µm and 2000 µm, respectively, where are hereafter labeled D600 and D2000. The actual sphere radius of curvature was measured with an AFM (Dimension 3100, Veeco, Plainview, NY, USA) to calculate the exact tip area functions. Some tests were also performed with a diamond Berkovich tip. The calibration of the tip area function was made using a standard fused silica sample. Indentations were carried out under a constant strain rate of 0.1 s^−1^ to a maximum depth of 5 µm for the Berkovich tip and 20 µm for the spherical tips. The load was also maintained at its maximal value for 10 s before unloading the material. Temperature and relative humidity conditions were set to 20 °C with RH varying from 30% to 45%.

The determination of the contact point was made using the method described by Moseson et al. [33] and successfully applied to porous ceramics [29,34]. *E* was determined using both CSM and Oliver and Pharr method [35]. The Poisson’s ratio, ν, was set to 0.2, which was a value obtained by Sanahuja et al. for highly porous materials, independently of the Poisson’s ratio of the solid matrix [19]. Three different samples for each silica were tested with a minimum of six indents per sample. To avoid proximity effects, the minimal center-to-center distance between two neighbor indents was set at four times the radius of contact for spheres and four times the side of residual imprints for the Berkovich tip. The observation of the residual imprints was found to be difficult due to the large radius of curvature of the spheres used and the uniform white color of the samples. Gold coating prior to SEM or optical microscopy observation was needed.

A spherical indentation test enabled the determination of contact stress–strain behavior [21,28,36] by plotting the mean pressure of hardness *p_m_*, which was defined as: $p_{m}=\frac{P}{\pi a^{2}}$ as a function of the indentation strain *a/R*, where *a* is the radius of contact and *R* is the sphere radius. As a spherical tip avoids stress singularities, an elastic contact is likely to occur at low loads. The theory of elastic contact, as suggested by Hertz, prescribes a linear relation between *p_m_*, the indentation stress, and *a/R*, the indentation strain: $p_{m}=\frac{4E_{r}}{3\pi }.\frac{a}{R}$ where *P* is the indentation load, and *E_r_* is the reduced modulus defined by: $\frac{1}{E_{r}}=\frac{1-\nu_{m}^{2}}{E_{m}}+\frac{1-\nu_{i}^{2}}{E_{i}}$, where *E* and *ν* are the Young’s modulus and Poisson’s ratio of the tested material and of the indenter. This usually corresponds to the initial stage of the stress–strain curves in real experiments [36] and permits determining the Young’s modulus *E* of the material (hereafter referred as the Hertz method for the determination of *E*).

Another parameter of interest is the ratio of the area under the unloading curve to the area under the loading curve, defined as the elastic-to-total work ratio *W_e_/W_tot_*. This ratio ranges from zero (totally irreversible behavior) to one (totally elastic behavior).

## 3. Results

**Figure 1:**
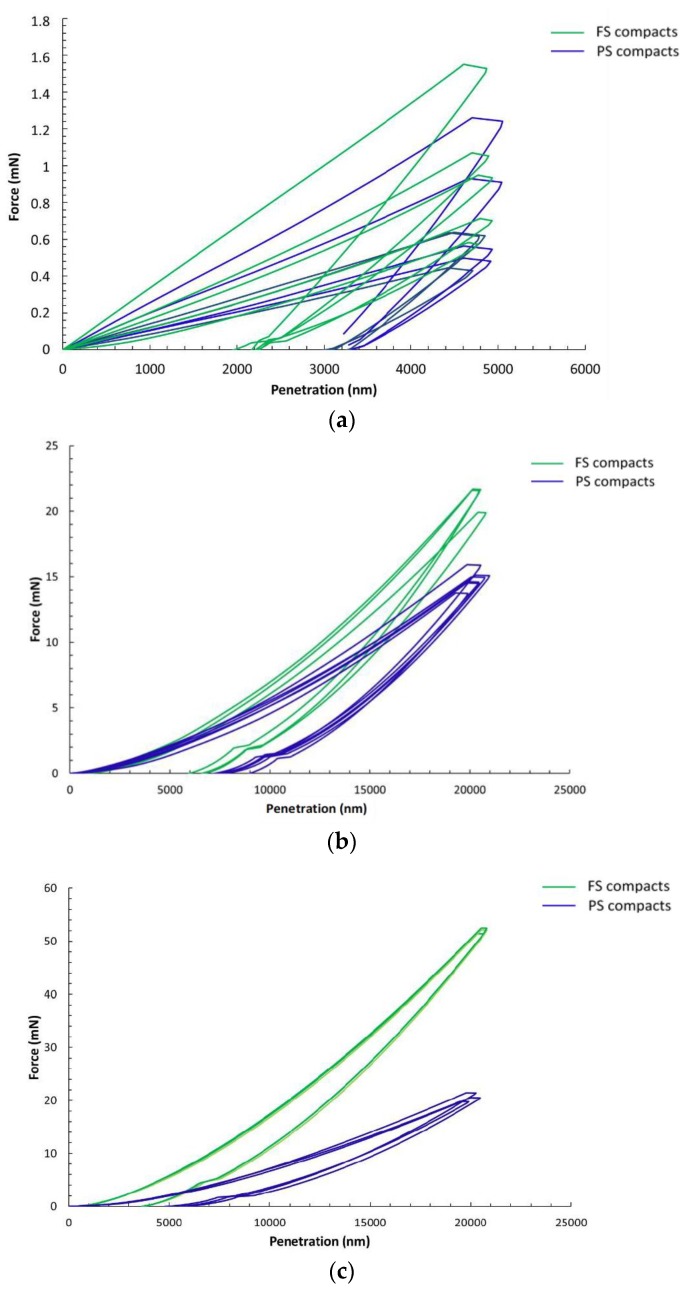
Load–deflection curves in spherical indentation on fumed silica (FS, in green) and precipitated silica (PS, in blue) compacts. (**a**) Berkovich tip; (**b**) 600-µm diameter sphere; and (**c**) 2000-µm diameter sphere.

The load–displacement curves measured on FS and PS samples using Berkovich, D600, and D2000 spheres are shown in Figure 1. The curves measured with Berkovich tips (Figure 1a) show a large scattering for both of the tested materials. Tests with D600 and D2000 (Figure 1b,c) spheres led to a much better repeatability of the load–displacement curves. Then, a clear distinction can be made between FS and PS compacts with a higher hardness for FS pellets as compared with PS, which was emphasized in the tests with D2000 spheres. The comparison of load–displacement curves for the three different tips shows a large influence of tip characteristics on the residual penetration depth after unloading (Figure 1). The Berkovich test led to larger irreversible fractions, which were illustrated by a large residual penetration depth compared with the maximum penetration depth. The irreversible energy fractions were lower in tests with D600 spheres, and even lower in tests with D2000 spheres. The latter show a strong elastic behavior, as illustrated by the evident elastic recovery during unloading with a limited residual penetration depth.

**Figure 2:**
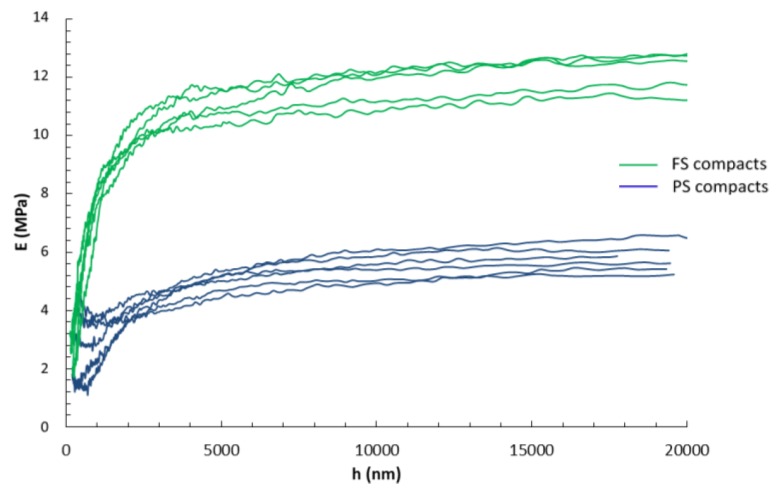
Young’s modulus as measured by CSM versus penetration depth with a 2000-µm diameter sphere for FS (in green) and PS (in blue) compacts.

The Young’s modulus versus penetration depth (CSM mode) in the D2000 tests is shown in Figure 2. The average modulus was extracted in the plateau region, which was above 5 µm of penetration depth, thus avoiding the influence of the surface roughness of the samples. The higher rigidity of the FS compacts as compared with PS ones was clearly demonstrated. 

**Figure 3:**
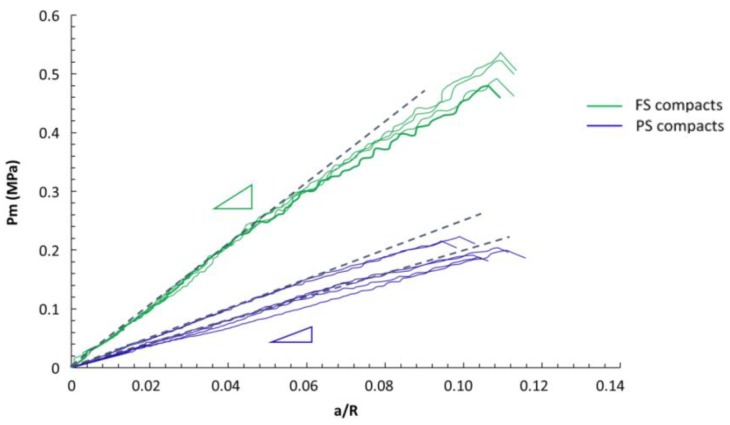
Contact stress–strain curves on FS (in green) and PS compacts (in blue) measured with a 2000-µm diameter sphere. Triangles illustrate the difference in slope between FS and PS compacts.

As the spherical indentation tests did not lead to a unique hardness value, contact stress–strain curves were plotted from the D2000 tests and are shown in Figure 3. They show a first linear relationship between hardness and *a/R*, allowing the determination of Young’s modulus by the Hertz method, followed by a non-linear increasing part. Then, the average pressure at the beginning of non-linearity could be extracted. The parameters for FS and PS pellets determined from instrumented indentation tests, including Young’s modulus, hardness, the elastic-to-total work ratio, are presented in Table 2 and Table 3.

The Young’s modulus determined with the CSM, Oliver and Pharr, and Hertz methods led to similar values for spherical indentation tests for both FS and PS pellets (Table 2). E values were slightly dependent on the size of spheres; they were higher with D2000 as compared with D600 for the FS samples, and higher with D600 for the PS samples. The strong influence of using a sharp tip as compared with a spherical one was noted, with much larger values of E for FS and PS compacts using a Berkovich tip. Comparing the two materials, FS compacts were approximately twice as stiff as PS from the D2000 tests, and only 25% higher from tests using D600 spheres. Berkovich indentation led to similar values of Young’s modulus for the two silica samples, showing even slightly higher values for PS compacts.

In terms of hardness, tests with Berkovich tips led to much higher values as compared with spherical tests. As spherical tests led to non-unique hardness values, the hardness indicated in Table 3 for the spherical tests corresponds to the pressure at the end of the elastic domain in the contact stress–strain curves (see Figure 3). Tests with D600 and D2000 spheres led to slightly different values of pressure for PS compacts, whereas for FS compacts, the values were identical. The comparison between FS and PS confirmed the large difference in properties already noted for the Young’s modulus, with a higher hardness for FS compacts, which was particularly evidenced for tests with D2000 spheres (FS pellets more than twice as hard as PS ones), and slightly less pronounced for tests with D600 spheres and Berkovich tips (FS pellets 40% harder than PS ones).

The elastic-to-total work ratio was largely dependent on the tip geometry, with the largest values for the D2000 sphere, and lowest values for the Berkovich tip. This is clearly illustrated in the load–displacement curves (Figure 1) by the ratio of areas under the unloading and under the loading curves, respectively. Regarding the two materials tested, FS compacts showed a higher elastic-to-total work ratio as compared with PS compacts, whatever the testing conditions.

**Figure 4:**
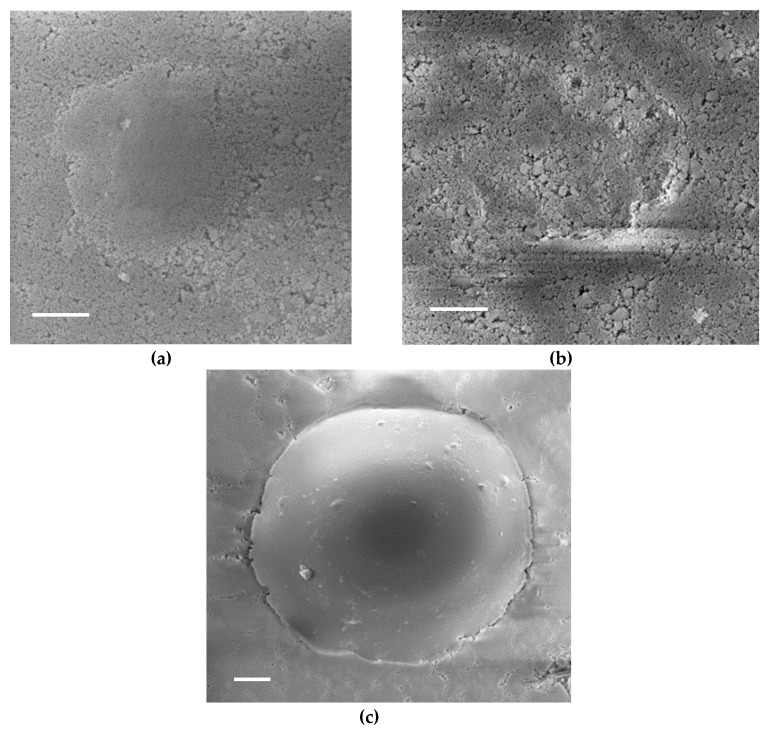

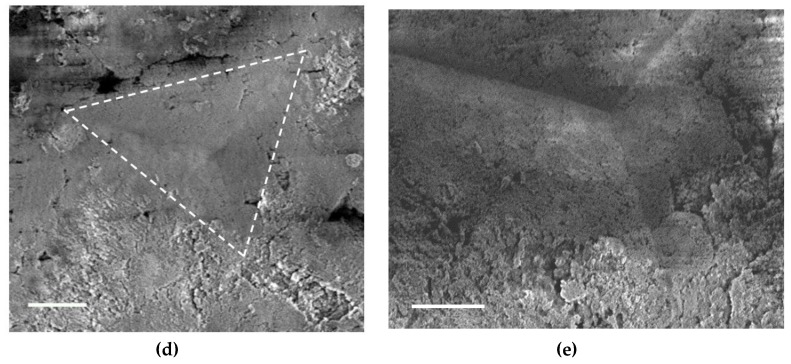
Images of residual indents: (**a**) D600 sphere on an FS compact, load 6 mN, (**b**) D600 sphere on a PS compact, load 6 mN, (**c**) D2000 sphere on an FS compact, load 50 mN, (**d**,**e**) Berkovich on an FS compact, load 1 mN (dotted lines in d added as guidelines). Scale bar: (**a**) 20 µm, (**b**) 20 µm, (**c**) 20 µm, (**d**) 20 µm, and (**e**) 10 µm.

Figure 4 illustrates the residual imprints observed with SEM. The high level of porosity gives a heterogeneous surface aspect with some charging effect, making the observation delicate, especially for tests with D2000 with a strong elastic recovery at unloading and a small residual depth. No pile-up was noted around the residual imprints, whatever the tip geometry. No macrocracks were found at the corner of Berkovich residual imprints (Figure 4d,e). Spherical imprints did not show macrocracks; however, ring cracks could be noted above a threshold load (approximately 10 mN for tests with D600) on FS samples (Figure 4c), but not on PS samples.

## 4. Discussion

### 4.1. Porous Silica Compacts, A Peculiar Material

The mechanical testing of highly porous silica compacts is a tough task due to the difficulty of manipulating samples with a very low stiffness and hardness. Instrumented indentation offers an advantage over standard mechanical tests (compression, bending) due to the possibility to test samples with non-standard geometry, requiring only two parallel and flat surfaces. However, the impossibility of mechanically polishing the surface without degradation (cracks, densification) requires the development of a specific preparation protocol. In this study, samples were processed by oedometric compression with a one-hour stress relaxation stage between loading and unloading to decrease the surface roughness of the sample. Preliminary tests (not shown) have shown that mean values of Young’s modulus and hardness are not affected by the relaxation stage, but that the experimental scatter was largely reduced after relaxation. The relaxation stage did not generate any dense “skin” layer at the sample’s surface, as illustrated in Figure 2 by the constant value of Young’s modulus versus penetration depth once the contact has been established. The white mat surface aspect of the samples, as well as their high porosity level, made the observation of residual imprints difficult with surface charging even after metal coating (Figure 4).

No piling up was noted neither in the spherical nor in the Berkovich tests, as already encountered on highly porous ceramics [28], which was related to the densification capacity of the material due to its high porous fraction. The densification of silica pellets in oedometric compaction at increasing pressures (0.15 to 1.2 MPa) has already been noted for FS samples and PS samples [11], [37]. Some circumferential cracks could also be noted on FS compacts on the periphery of residual imprints for spherical tests for the highest loads used in this work (Figure 4). No macrocrack propagation from the corners of Berkovich residual imprints was noted, which can also be related to the densification of the material under the indent, reducing the residual tensile stress field at the origin of crack propagation after unloading [37]. This lower sensitivity to crack propagation from indents in highly porous ceramics has already been noted [38,39].

### 4.2. Influence of the Tip Geometry

The mechanical parameters extracted from the instrumented indentation tests, hardness, and Young’s modulus varied largely with the tip characteristics, which included the geometry (sharp or spherical) and size for spherical tips.

Tests with Berkovich tips led to much higher *E* and *H* values than with spherical tips (Table 2 and Table 3) as well as to a lower elastic contribution, as testified by the lower elastic-to-total work ratio (Figure 1, Table 3). Irreversible phenomena below the indenter were favored by the high stress concentration generated by Berkovich tips, as compared with spherical tips.

Comparing the spherical tests with D600 and D2000 spheres, a small difference was noted in the pressure at the end of the elastic domain of the contact stress–strain curves for PS compacts only, with average values of 0.15 MPa and 0.25 MPa for D2000 and D600, respectively (Figure 3, Table 3). This may be related to a different stress field under the indenter for a given *a/R* value between the two sphere sizes for a damageable material, as already noted in the literature on porous ceramics and mineralized bone tissue [29,39]. Nevertheless, it should be noted that spherical tests are much less sensitive to damage than the Berkovich test, which is due to the smoother stress fields as compared with sharp indenters [31,39].

The samples tested in this work were highly porous (90% of pores) and displayed a granular structure, which was made of a stacking of nanosized silica particles. The presence of an elastic domain for such material may be questioned. From the results of this work, it appears that a Young’s modulus can be determined from spherical indentation tests. Indentation tests with a given sphere diameter gave similar values for *E* with different methods (Table 2), which included the Hertz and Oliver Pharr methods based on loading and unloading curves recorded at a static strain rate, and the CSM method on a high-frequency measurement, thus validating the existence of an elastic domain for both FS and PS compacts in ambient environmental conditions. A small difference was noted when comparing the Young’s modulus extracted from D600 and D2000 tests, with a lower value for the FS modulus and a higher value for PS with D600 as compared with D2000. It seemed that tests with the D600 sphere showed a more pronounced tendency to involve irreversible mechanisms as compared with tests with the D2000 sphere, as testified by the lower elastic-to-total work ratio (Table 3). As a consequence, the Young’s modulus calculated from the D600 tests may be influenced by damage, as noted to a larger extent on tests with Berkovich tips. Finally, D2000 spheres appear to be the preferred geometry to characterize compacts of silica nanopowders.

### 4.3. Comparison between Fumed and Precipitated Silica

From the experimental data collected in this work, a large difference in terms of mechanical properties is evidenced between compacts of FS and PS silica with a similar total fraction of pores. The comparison of samples is made at a similar apparent density, as density is a first-order parameter on the properties of porous materials [12,40]. FS compacts offered higher rigidity and hardness as compared with PS compacts, with a Young’s modulus of 12 MPa and an average pressure at the end of elastic domain of 0.35 MPa versus 6 MPa and 0.15 MPa respectively (data from D2000 tests). This is consistent with previous work on the formulation of the VIP core [7] that showed the superior mechanical behavior of FS materials over PS in VIP core formulations. It is also consistent with the observation during handling that FS pellets are stronger than PS pellets, which tend to collapse under finger pressure. 

The larger rigidity and pressure at the end of the elastic domain of FS compacts (more than twice the value of PS compacts) may be explained by the structural organization of the stacking of silica powders. The multi-scale organization of similar compacts was characterized by transmission electron microscopy and by small angle X-ray scattering (SAXS) experiments [11]. The organization at the submicron-length scale appeared as clearly different between FS and PS compacts. Silica agglomerates of FS silica were shown to have a high aspect ratio, which was linked with a stronger entanglement capacity, and finally with a stronger material as a given total porosity fraction [12,41]. When comparing the hardness and rigidity of silica pellets, it has to be noted that FS and PS samples were compacted at two different pressures (0.6 MPa for FS and 0.15 MPa for PS) to prepare samples with a similar apparent density. The different compaction behavior between FS and PS powders can also be related to the stronger entanglement between agglomerates of silica particles in FS samples as compared with PS ones. Compaction pressures are of the same order of magnitude as the mean pressures at the end of elastic domain measured with spherical indentation (0.3 MPa for FS and 0.15 MPa for PS). However, it needs to be noted that the stress field in oedometric compaction and spherical indentation tests are different, and a direct comparison of the pressure level is not possible.

When tested with Berkovich tips, smaller differences in *E* and *H* values were noted between FS and PS samples as compared with spherical tests (Table 3). This has to be related to the large irreversible phenomenon contribution, and mainly to a strong densification under the indenter, as testified by the large values of *E* and *H* with sharp tip as compared with spheres. It seems that once the stacking of silica agglomerates locally collapses and subsequently densifies, the differences between FS and PS samples are greatly reduced. It also appears that *E* measured using a Berkovich tip was not representative of the virgin material, showing the strong influence of the densification phenomena or damage. This tendency was already noted for bone tissue, which is a damage-sensitive material [39].

### 4.4. Comparison with Other Porous Silica Samples and Silica Aerogels

As mentioned in the introduction, no previous indentation tests on compacts of silica nanopowders were found in the literature. Instrumented indentation tests were carried out on silica aerogels [20,21,25] and foamed silica [42], which had apparent densities close to those of the silica compacts tested in this work.

The instrumented indentation of silica macroporous scaffolds with a solid fraction of 18 vol % and 24 vol % have shown that the major mechanism occurring during indentation is densification by local fracture of the solid walls between pores [42], with a very limited elastic contribution in tests with both Berkovich and spherical tips. Silica aerogels with a similar solid fraction as the silica compacts tested in this study (apparent density of 340 g/m^3^ i.e., a solid fraction 15 vol % [21]) were also tested using Berkovich and spherical tips at room temperature. Young’s modulus and hardness measured with both tip geometries led to similar values i.e., 110 MPa for *E* and 10 to 11 MPa for *H*. These *E* and *H* values of silica aerogels were larger than those noted for the silica compacts tested in this study (Table 3). The mechanism involved during the indentation of silica aerogels is the bending of nanoligaments of silica with no major signs of densification, but with the presence of cracks from the corner of the residual imprint in Berkovich tests, and of numerous ring cracks in spherical tests [21]. The 3D distribution of the solid phase in aerogel and silica compacts is different, with a 3D network of small size (3 to 6-nm) silica ligaments for aerogels [43,44] versus a multi-scale stacking of aggregates and agglomerates for silica compacts [11]. The former give a pore size distribution centered on 10 nm, while the latter give a multi-scale pore distribution with pores from several nanometers to tens of micrometers [11]. The stronger resistance to the densification of silica aerogels as compared with silica compacts may be related to the different organization of the solid phase and to the presence of Si–C bonds, leading to a better strength and a higher amount of stored elastic energy, and therefore to a more brittle behavior.

### 4.5. Final Remarks, Outlook

A fine characterization of the mechanical properties of VIP core is of critical importance, as these materials need to show sufficient mechanical properties to enable the fabrication of panels at the lowest possible density in order to limit the thermal conductivity. It appears from this study that indentation testing with a large sphere offers the best choice to extract elastic properties and estimate the average pressure at the end of elastic domain with a large tested volume.

However, the characterization of the damaged volume below the residual indent needs to be improved, possibly through using FIB slice and viewing the observations. In particular, the quantification of densification would bring valuable information for identifying the constitutive law of the material, as already demonstrated for densifying glasses or porous ceramics [29,45,46]. Modeling the mechanical properties of a multi-scale stacking of silica particles [43] also appears as critical to better understand the structure–properties relationship of silica compacts, similarly to that which has been done for silica aerogels [44].

We have chosen in this work to maintain limited and similar environmental conditions by working in an ambient environment under controlled temperature and with only small variations in relative humidity. Large differences exist in the hydrophilicity of silica nanopowders, which are related to their processes and possible surface treatments (see Table 1 [5,47]). Thus, an influence of the water absorption on the mechanical properties of compacts is expected. It could be useful to test the compacts at 140 °C to eliminate the physisorbed water [48,49], and therefore get closer to the conditions in the core of a VIP. At the same time, it is also of importance to characterize the aging of silica compacts versus relative humidity, as the pressure inside the VIP is known to increase during the service life of the insulating materials [49].

## 5. Conclusions

In this work, we have demonstrated the relevance of spherical indentation to characterize the mechanical properties of highly porous silica compacts with a porosity fraction of 90% and a very low rigidity, with a Young’s modulus ranging between 5–10 MPa. This experimental technique allows discriminating the properties of two types of silica powder (fumed and precipitated) on compacts with a similar total volume fraction of porosity. Compacts of fumed silica are approximately twice as stiff and harder as those of precipitated silica. The behavior of porous silica compacts under indentation is shown to be elastic damageable with a notable influence of the tip geometry on the measured properties. Tests with large spheres are shown to be preferable over sharp tips for estimating the average properties of porous silica compacts.

## Figures and Tables

**Table 1:** Main physical characteristics of fumed silica (FS) and precipitated silica (PS) compacts (average values and standard deviations when available) as determined in this work. Water uptake is measured on silica powders; after drying at 140 °C for 1 h, skeletal density is measured with a helium pycnometer (Accu-Pyc, Micrometritics, Norcross, GA, USA), and specific surface by nitrogen absorption (Belsorp-max, BEL, Germany). Numbers in italic stand for the standard deviation.

Silica	Solid Fraction	Specific Surface	Skeletal Density	Apparent Density	Water Uptake (wt %)
	%	g/m^2^	kg/m^3^	kg/m^3^	24 °C 45% RH
FS	9.9	187 *_15_*	2200	218 *_2_*	0.5 *_0.1_*
PS	9.4	207 *_20_*	2100	198 *_2_*	6.0 *_0.4_*

**Table 2:** Average values and standard deviations of elastic modulus of FS and PS compacts determined from indentation tests with different tips and using different calculation methods. Poisson’s ratio is considered equal to 0.2. CSM: continuous stiffness measurement, OP: Oliver–Pharr. Numbers in italic stand for the standard deviation.

E (MPa)	Sphere						Berkovich
	D2000			D600			
	*CSM*	*OP*	*Hertz*	*CSM*	*OP*	*Hertz*	*CSM*
FS	11.8 *_0.6_*	12.6 *_0.6_*	12.3 *_1.0_*	10.0 *_0.6_*	10.7 *_0.6_*	10.6 *_1.0_*	21 *_10_*
PS	5.5 *_0.4_*	6.1 *_0.4_*	5.7 *_1.0_*	8.2 *_0.3_*	9.1 *_0.3_*	7.1 *_1.0_*	24 *_10_*

**Table 3:** Average values and standard deviations of elastic modulus, hardness, and elastic-to-total work ratio determined from indentation tests with spherical and Berkovich tips, using the CSM method. The values of hardness for the D2000 and D600 spherical tests correspond to the end of the elastic domain in the contact stress–strain curves. Numbers in italic stand for the standard deviation.

Property	Sample	Sphere		Berkovich
		D2000	D600	
E (MPa)	FS	11.8 *_0.6_*	10.0 *_0.6_*	21 *_10_*
	PS	5.5 *_0.4_*	8.2 *_0.3_*	24 *_10_*
H (MPa)	FS	0.35 *_0.01_*	0.35 *_0.02_*	5.2 *_2.4_*
	PS	0.15 *_0.01_*	0.25 *_0.02_*	3.8 *_1.8_*
W_e_/W_tot_	FS	0.77 *_0.02_*	0.65 *_0.03_*	0.45 *_0.08_*
	PS	0.65 *_0.02_*	0.55 *_0.03_*	0.33 *_0.06_*
